# Exploring the Impact of Hydrolyzed Collagen Oral Supplementation on Skin Rejuvenation: A Systematic Review and Meta-Analysis

**DOI:** 10.7759/cureus.50231

**Published:** 2023-12-09

**Authors:** Dian Andriani Ratna Dewi, Abraham Arimuko, Lilik Norawati, Satya W Yenny, Nenden L Setiasih, Angki Perdiyana, Nabila Arkania, Farrasila Nadhira, Ni Wiliantari

**Affiliations:** 1 Department of Dermatovenereology, The Republic of Indonesia Defense University, Bogor, IDN; 2 Department of Dermatovenereology, Gatot Soebroto Army Hospital, Central Jakarta, IDN; 3 Department of Dermatovenereology, Faculty of Medicine, Andalas University, Padang, IDN; 4 Department of Dermatovenereology, Faculty of Medicine, Yarsi University, Central Jakarta, IDN; 5 Department of Dermatovenereology, Faculty of Military Medicine, The Republic of Indonesia Defense University, Bogor, IDN; 6 Department of Dermatovenereology, Faculty of Medicine, Public Health, and Nursing, Gadjah Mada University, Special Region of Yogyakarta, IDN; 7 Department of Dermatovenereology, Ratna Dewi Principal Clinic, Bekasi, IDN

**Keywords:** skin hydration, revitalizing, skin elasticity, skin moisture, skin aging, dietary supplements, hydrolyzed collagen, skin rejuvenation

## Abstract

With increasing life expectancy, the quest for skin rejuvenation has gained prominence among individuals of diverse age groups. The popularity of nutricosmetics, notably dietary supplements, has garnered significant attention in recent years. Many scientific investigations have amassed compelling evidence highlighting the positive impact of hydrolyzed collagen supplementation in mitigating the visible signs of skin aging. This study aims to know the powerful effect of hydrolyzed collagen on the skin. This research method is to conduct a systematic review followed by a meta-analysis of the clinical trial focusing on randomized, double-blind, and controlled trials that examined the oral consumption of hydrolyzed collagen and reported outcomes related to skin aging, wrinkles, moisture levels, elasticity, and firmness. The selected articles from CENTRAL, PubMed, Google Scholar, and ScienceDirect databases were published from 2017 to 2023. The subsequent meta-analysis, comprising 14 distinct studies and a collective cohort of 967 participants, revealed encouraging findings favoring hydrolyzed collagen supplementation. It consistently demonstrated substantial enhancements in skin moisture levels and elasticity compared to the placebo group, a trend robustly corroborated by subgroup analysis. These compelling findings underscore the effectiveness of a 12-week regimen of hydrolyzed collagen supplementation in revitalizing the skin by augmenting its hydration and elasticity.

## Introduction and background

Collagen is a vital structural protein found in diverse connective tissues like skin, tendons, cartilage, and bone, making up a substantial fraction, roughly 25-30%, of the total proteins in the human body [1]. Within the skin, collagen works in concert with other components, including hyaluronic acid, reticulin, and elastin, to establish a vital framework for diverse types of skin cells, including fibroblasts, keratinocytes, melanocytes, and specialized immune cells [2]. This complex network assumes a fundamental role in furnishing mechanical support and preserving the overall structural integrity of the skin [1,3].

Skin aging is a natural progression influenced by a blend of internal alterations and external elements that lead to damage, with most of these modifications occurring in the dermis [1,4]. Elastic fibers, comprising a core of elastin intertwined with microfibrils primarily made of fibrillin, represent another crucial element in the dermis. These fibers play a role in enhancing the skin's elasticity, resilience, and tensile strength. Moreover, significant alterations occur in the epidermis, the most prominent of which is the accumulation of corneocytes, leading to a coarse and dull appearance of the skin. Furthermore, skin aging also entails diminishing cutaneous blood vessels and structural modifications in the subcutaneous tissue [4,5].

In addition, the natural aging process also leads to decreased collagen production, resulting in visible signs of aging, such as reduced elasticity and reduced hydration [2]. Hydrolyzed collagen (HC), derived from the enzymatic breakdown of native collagen, has been introduced as an additional approach to rejuvenate the skin [6]. HC is rich in amino acids such as hydroxyproline, proline, and glycine, which are absorbed as dipeptides and transported to the skin. These dipeptides enhance the bioactivity of dermal fibroblasts by stimulating collagen synthesis, thereby improving skin moisture levels and elasticity [1,6].

With the growing body of scientific literature and clinical investigations addressing collagen supplementation continuing to expand globally, there is an apparent demand to collect and scrutinize this information to aid informed decision-making regarding supplementation [1,7]. Hence, the main aim of this research was to gather and synthesize the existing knowledge regarding the effects of HC supplementation on human skin. This goal was accomplished by conducting a systematic review followed by a meta-analysis of clinical trials that specifically addressed the process of skin rejuvenation.

Materials and methods

Our systematic review and meta-analysis research followed the specific guidelines outlined in PRISMA (Preferred Reporting Items for Systematic Reviews and Meta-Analyses) and adhered to the methodology described in the Cochrane Handbook for Systematic Reviews of Interventions, with the precise version being 6.3.2022.

Eligibility Criteria (Inclusion and Exclusion Criteria)

Before commencing the literature search, we established a set of criteria for inclusion and exclusion to determine the relevancy of the data. The inclusion criteria encompassed the following aspects: (1) randomized studies, which encompassed randomized controlled trials and clinical trials; (2) participants in good health; (3) interventions involving HC; (4) evaluation of patient skin hydration, skin elasticity, wrinkles, transepidermal water loss (TEWL), firmness, and skin radiance as outcomes; and (5) published articles in the English language.

On the other hand, the exclusion criteria comprised (1) participants below 20 years, (2) patients with skin conditions, and (3) research written in a language other than English. Retrieved studies were selected by two independent reviewers (AP and DD), who initially evaluated the appropriateness of the title and abstract. Afterward, different reviewers confirmed the suitability of the full text. In situations where disagreements arise, input from other authors (LN and MW) was sought to reach a consensus.

Standard of References

We referred to a randomized controlled trial that investigated the impact of oral administration of HC on skin hydration and elasticity in a group of healthy individuals.

Search Strategy

As indicated in the initial attachments, we conducted our literature research by utilizing the following databases: CENTRAL (Cochrane Library), PubMed, Google Scholar, and ScienceDirect, published in 2017-2023. These searches were performed between November 4 and November 6, 2023. All search terms were aligned with the MeSH (Medical Subject Headings) browser. We incorporated keywords in the search field along with Boolean operators. The specific keywords were chosen in line with the guidelines for Boolean operator keywords, structured as follows: (((Hydrolyzed collagen [MeSH Terms]) AND (Skin rejuvenation OR skin aging [MeSH Terms])) AND (Skin elasticity [MeSH Terms])) AND (Skin hydration [MeSH Terms]).

Data Extraction

Two evaluators (AP and DARD) independently extracted and recorded the information in an electronic spreadsheet. The extracted data encompassed various details, including the total number of patients, their gender, age, the groups under investigation in each study (consisting of treatment and placebo groups), the duration of the study, the nature of the intervention administered, and the primary outcomes evaluated, with a specific emphasis on factors like skin hydration and elasticity. Furthermore, baseline data over the characteristics measured in the placebo and treatment groups were collected. All numerical data underwent analysis, including mean values and their corresponding standard deviations (SDs).

Analysis (Statistical, Sensitivity, and Assessment of Bias)

The studies were categorized based on the similarity of their outcomes, and for each cluster, separate meta-analyses were conducted. In the statistical analysis, the inverse variance method was employed to compute the mean difference and SD for continuous variables, with a significance threshold set at a probability (P) value less than 0.05 to establish statistical significance. The overall effect was presented as a z-value, accompanied by a 95% confidence interval (CI) for interpretation.

To assess the heterogeneity of results among the included studies, the Higgins I (I2) statistical model was used, where I2 values of 50% or less indicated low to moderate heterogeneity. In comparison, values exceeding 75% showed high heterogeneity. Subgroup analyses were conducted, stratified by units of measurement, to pinpoint sources of heterogeneity. Sensitivity analysis was also performed, excluding studies with unique measurement units or varying sample sizes to minimize the impact of potentially influential studies.

Publication bias was visually evaluated using a funnel chart. The quality of the studies was assessed by Cochrane guidelines, with each study being classified based on the five types of bias (selection, performance, detection, attrition, and reporting bias) as outlined in the Risk of Bias (RoB)-2 tool. All statistical analyses were carried out using RevMan version 5.4 (The Cochrane Collaboration, London, UK).

## Review

Study and characteristics results

**Figure 1:**
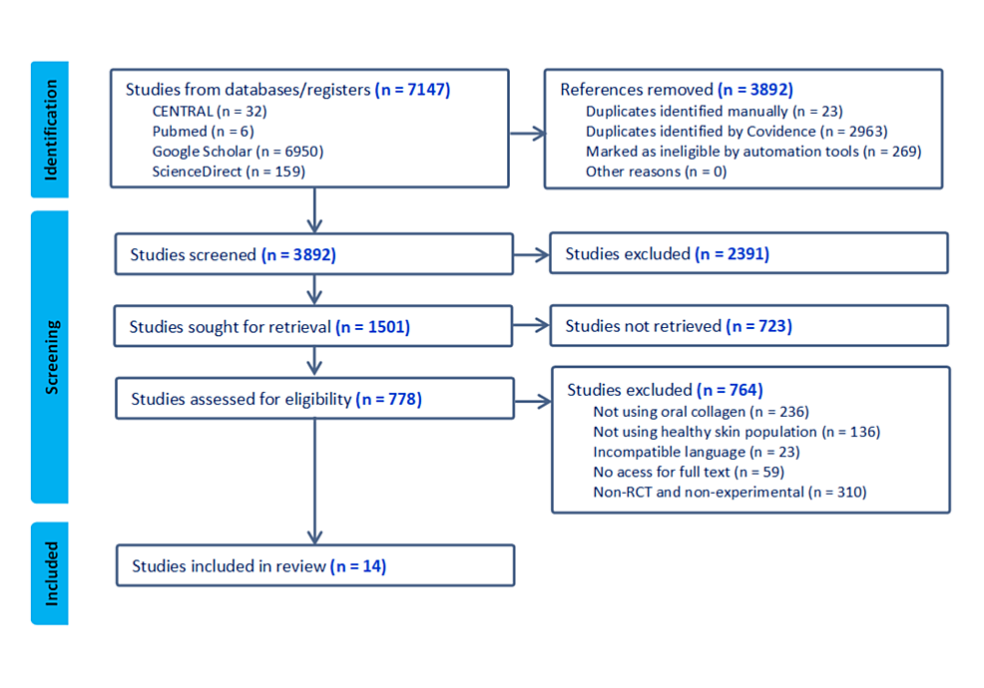
Flowchart of the included studies. RCT: randomized controlled trial. This figure was created by AP, one of the authors of this article.

The initial search produced 7,147 articles from the databases (Appendix). After applying predefined criteria in the title and abstract screening phase and removing duplicate entries, 21 articles were identified to examine their full texts thoroughly. Among these, seven articles were excluded based on the predetermined inclusion and exclusion criteria, resulting in a final selection of 14 articles that met the requirements for quantitative analysis. Figure 1 illustrates the study selection process, delineating the different phases of identification, selection, eligibility, and inclusion by the PRISMA guidelines for systematic review composition.

Study characteristics

This meta-analysis encompassed 14 randomized controlled trials (RCTs) involving 967 patients. The duration of HC supplementation in these studies varied from four to 12 weeks. The RCTs analyzed in this study were classified based on their impact on skin hydration and elasticity. Regarding skin hydration, five studies investigated collagen sourced from fish, while nine examined collagen from non-fish origins. Regarding skin elasticity, four studies concentrated on fish-derived collagen, and 10 studies explored collagen from non-fish sources. Comprehensive details regarding the characteristics of the RCTs included in this analysis can be found in Table 1.

**Table 1:** Description of the included studies. APCP: AP collagen peptide; HC: hydrolyzed collagen; TEWL: transepidermal water loss. This table was created by AP, one of the authors of this article.

No.	Author, year	Title (country)	Study design	Number of participants, n (HC/placebo)	Female/male	Intervention (supplement composition)	Duration of outcome measures	Outcome
1.	Bolke et al. (2019) [2]	Effects of a collagen supplement on skin aging and skin health: a randomized, placebo-controlled, blind study (Germany)	The clinical study employed a randomized, placebo-controlled, single-blind design	72 (36/36)	72/0	Collagen peptides and acerola fruit extract with endogenous antioxidants, such as vitamin C, zinc, biotin, and a native vitamin E complex	4 weeks	Hydration, elasticity, wrinkles, and skin density
2.	Czajka et al. (2018) [8]	Daily oral supplementation with collagen peptides combined with vitamins and other bioactive compounds improves skin elasticity and has a beneficial effect on joint and general wellbeing (UK)	A double-blind, randomized, placebo-controlled monocentric study	120 (61/59)	91/29	Hydrolyzed fish collagen type I (4,000 mg), molecular weight of 0.3–8 kDa, hyaluronic acid, glucosamine hydrochloride, L-carnitine, black pepper and maca extracts	12 weeks	Elasticity, biopsies, and self-perception questionnaire
3.	Evans et al. (2020) [9]	A randomized, triple-blind, placebo-controlled, parallel study to evaluate the efficacy of a freshwater marine collagen on skin wrinkles and elasticity (Canada)	A randomized, triple-blind, placebo-controlled study	50 (25/25)	50/0	Hydrolyzed collagen powder derived from *Pangasius hypophthalmus*, a tropical and sustainable freshwater fish	12 weeks	Wrinkles, elasticity, firmness, radiance, and self-reported appearance
4.	Genovese et al. (2017) [5]	An insight into the changes in skin texture and properties following dietary intervention with a nutricosmeceutical containing a blend of collagen bioactive peptides and antioxidants (Italy)	A double-blind, randomized, placebo-controlled clinical trial	120 (60/60)	111/9	High dosage of hydrolyzed collagen	12 weeks	Elasticity, biopsies, and subjective questionnaire
5.	Ito et al. (2018) [10]	Effects of composite supplement containing collagen peptide and ornithine on skin conditions and plasma IGF-1 levels—a randomized, double-blind, placebo-controlled trial (Japan)	Double-blind, placebo-controlled pilot study	21 (10/11)	17/4	Collagen peptide and ornithine (CPO)	8 weeks	Elasticity, moisture, TEWL, spots, wrinkles, and skin pores
6.	Jung et al. (2021) [11]	Oral intake of enzymatically decomposed AP collagen peptides improves skin moisture and ceramide and natural moisturizing factor contents in the stratum corneum (Korea)	Double-blind, randomized, parallel, and placebo-controlled study	50 (25/25)	28/22	1000 mg APCP (a type of collagen peptide)	12 weeks	Skin hydration, TEWL, skin gloss, and flexibility
7.	Kim et al. (2018) [12]	Oral intake of low-molecular-weight collagen peptide improves hydration, elasticity, and wrinkling in human skin: a randomized, double-blind, placebo-controlled study (Korea)	A randomized, double-blind, placebo-controlled study	64 (33/31)	64/0	Low-molecular-weight collagen peptide	6 & 12 weeks	Skin hydration, wrinkles, and elasticity
8.	Miyanaga et al. (2021) [13]	Oral supplementation of collagen peptides improves skin hydration by increasing the natural moisturizing factor content in the stratum corneum: a randomized, double-blind, placebo-controlled clinical trial (Japan)	A randomized, double-blind, placebo-controlled clinical trial	97 (62/33)	97/0	Collagen peptides 1 gram and 5 gram	12 weeks	Skin water content, TEWL, elasticity, and thickness
9.	Nomoto et al. (2020) [14]	Effect of an oral nutrition supplement containing collagen peptides on stratum corneum hydration and skin elasticity in hospitalized older adults: a multicenter open-label randomized controlled study (Japan)	A multicenter open-label randomized controlled study	39 (20/19)	27/12	10.0 g of collagen peptides	2-8 weeks	Stratum corneum hydration and skin elasticity
10.	Ping et al. (2020) [15]	Collagen formula with Djulis for improvement of skin hydration, brightness, texture, crow’s feet, and collagen content: A double-blind, randomized, placebo-controlled trial (Taiwan)	A double-blind, randomized, placebo-controlled trial	50 (25/25)	50/0	Collagen and Djulis	8 weeks	Skin hydration, brightness, texture, and crow’s feet
11.	Sangsuwan et al. (2020) [16]	Four-weeks daily intake of oral collagen hydrolysate results in improved skin elasticity, especially in sun-exposed areas: A randomized, double-blind, placebo-controlled trial (Thailand)	A randomized, double-blind, placebo-controlled trial	38 (17/19)	38/0	Collagen hydrolysate (CH) derived from fish scale and skin	4 weeks	Elasticity
12.	Schwartz et al. (2019) [17]	Novel hydrolyzed chicken sternal cartilage extract improves facial epidermis and connective tissue in healthy adult females: a randomized, double-blind, placebo-controlled trial (USA)	A randomized, double-blind, placebo-controlled trial	128 (65/63)	128/0	300 mg of hydrolyzed collagen type-II (HC-II), 100 mg of glycosaminoglycan (GAG), chondroitin sulfate (CS), and 50 mg of hyaluronic acid (HA)	12 weeks	Erythema, hydration, TEWL, elasticity, wrinkles, dermal collagen, and subjective questionnaire
13.	Tak et al. (2021) [18]	Effect of collagen tripeptide and adjusting for climate change on skin hydration in middle-aged women: a randomized, double-blind, placebo-controlled trial (Japan)	A randomized, double-blind, placebo-controlled clinical trial	84 (42/42)	82/0	Collagen tripeptide	12 weeks	Hydration, elasticity, and wrinkles
14.	Zmitek et al. (2020) [19]	Effects of a combination of water‐soluble coenzyme Q10 and collagen on skin parameters and condition: results of a randomised, placebo‐controlled, double‐blind study (Slovenia)	A randomized, placebo‐controlled, double‐blind study	34 (17/17)	34/0	Per 10 mL: hydrolyzed fish collagen: 4000 mg, water‐soluble CoQ10: 50 mg, vitamin C: 80 mg, vitamin A: 920 μg, biotin: 150 μg)	12 weeks	Dermal density, hydration, TEWL, wrinkles, moisture, and dermal microrelief
Total				967 (492/475)	891/76	Composition of participants: female (92%), male (8%), HC (51%), placebo (49%)		

The evaluation of skin hydration levels usually involves the application of a non-invasive instrument known as a corneometer. This device emits a high-frequency electrical current onto the skin's surface and quantifies the moisture content in the upper layers of the skin, expressed in corneometric units. The corneometer is widely employed to appraise topical products' effectiveness and gauge the skin's general condition by offering valuable insights into the skin's moisture barrier. Consequently, it is helpful for measuring skin hydration levels and appraising the efficacy of skincare products [2,7,10,12].

On the other hand, skin elasticity assessment frequently involves using cutometry, a non-invasive technique that offers valuable insights into skin health. This approach entails applying controlled negative pressure to a small skin area and measuring the resulting deformation, which is directly linked to skin elasticity. Cutometry finds extensive use in research and clinical contexts to evaluate skin elasticity levels and track the evolution of the skin's condition over time. Generally, it is a dependable and secure tool for skin health assessment [2,3,7,12,14,16,17].

Meta-analysis results

Pooled Analysis of Selected Studies

Several articles were excluded from our study for various reasons. These exclusions were a result of multiple factors, including some articles not conducting measurements for skin hydration levels and elasticity. Furthermore, specific articles needed more standard deviation data, provided adequate direct information regarding moisture and elasticity, or allowed for comparing their results with the intervention group. Consequently, these studies needed to meet the essential criteria necessary for their inclusion in our research. It is important to note that within the group of RCTs ultimately included in our analysis, patients were systematically divided into two groups based on their consumption of collagen supplements and subsequent skin hydration or elasticity measurements. This systematic categorization laid the foundation for the subsequent execution of a meta-analysis, enabling us to draw meaningful conclusions from the accumulated data.

Subgroup Analysis

**Figure 2:**
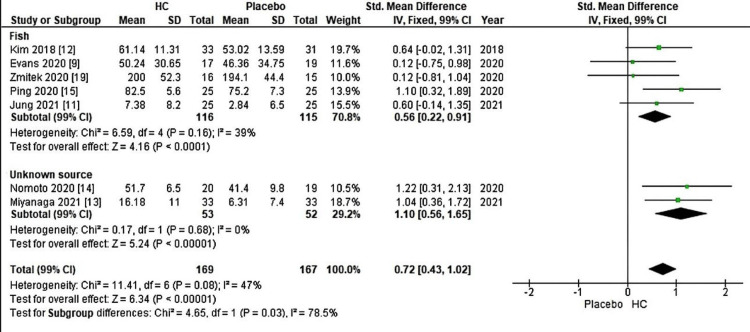
Forest plot of comparison: the comparison of skin hydration influenced by hydrolyzed collagen derived from fish and that is unknown (skin hydration, outcome: source). This figure was created by AA, one of the authors of this article.

**Figure 3:**
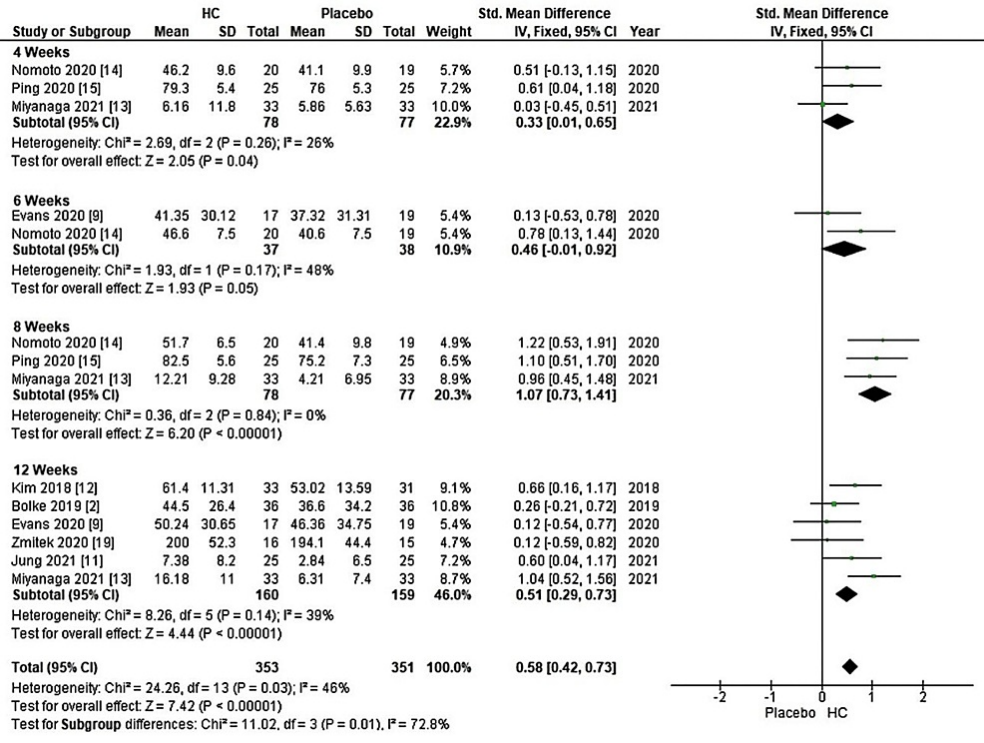
Forest plot of comparison: skin hydration, outcome: duration (weeks). HC: hydrolyzed collagen. This figure was created by AA, one of the authors of this article.

Collagen supplements come in various forms, such as gels, liquids, and capsules. The type of collagen used in these supplements can vary based on their source, with fish collagen being one of the most common types. In Figure 2, a forest plot displays a meta-analysis categorized by reference, including seven studies (five from fish sources and two from unknown sources). This meta-analysis focuses on combined skin hydration estimates comparing patients supplemented with HC to the placebo group. Notably, supplementation resulted in a significant increase, with a Z-value of 6.34 (P < 0.00001), supported by an overall effect size (95% CI) of 0.72 (0.43, 1.02). Additionally, Figure 3 presents a forest plot of a meta-analysis classified by the duration of the studies (four to 12 weeks) that assessed skin hydration across 14 studies. In this case, HC supplementation also significantly improved skin hydration compared to the placebo group, with a Z-value of 7.42 (P < 0.00001) and an overall effect size (95% CI) of 0.58 (0.42, 0.73).

**Figure 4:**
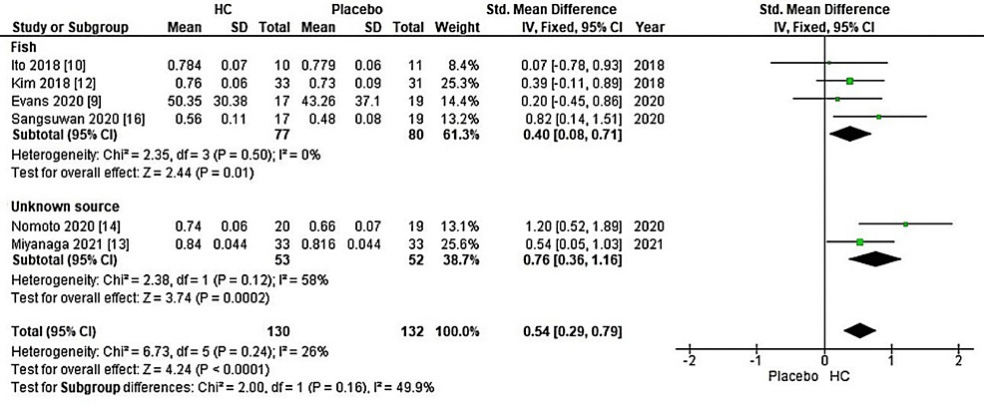
Forest plot of comparison: skin elasticity, outcome: source. HC: hydrolyzed collagen. This figure was created by LN, one of the authors of this article.

**Figure 5:**
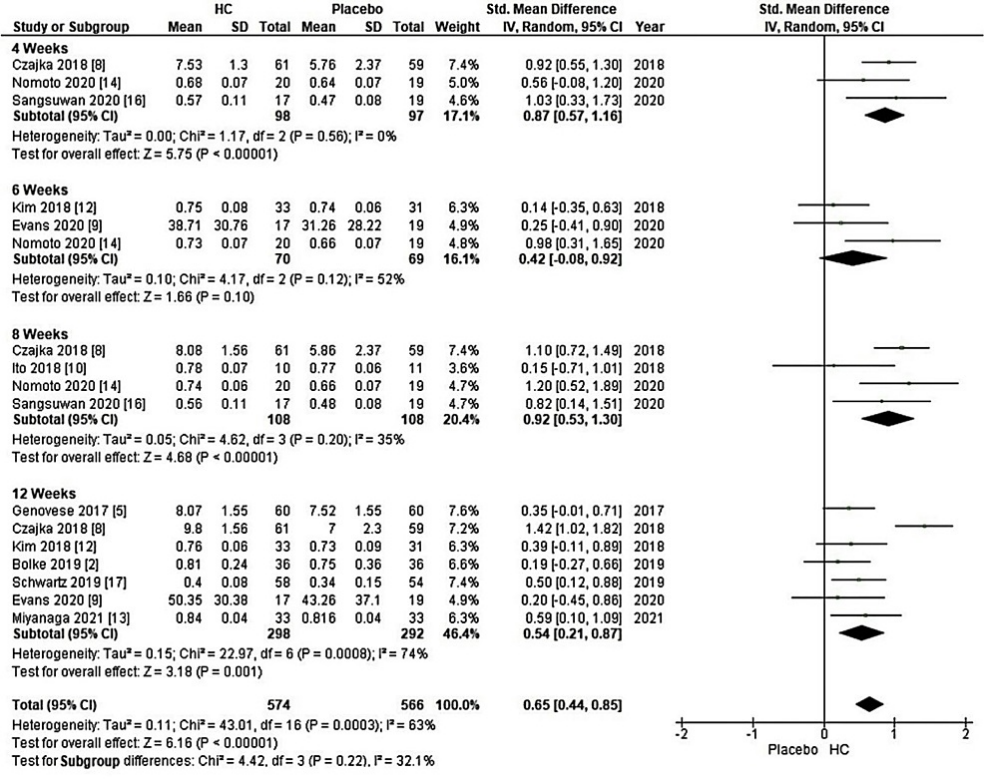
Forest plot of comparison: skin elasticity, outcome: duration (weeks). HC: hydrolyzed collagen. This figure was created by LN, one of the authors of this article.

Furthermore, the meta-analysis conducted three subgroup analyses to examine the impact of HC source and the duration of HC supplementation on skin elasticity, including measurements such as gross elasticity, net elasticity, and the elastic portion assessed using a cutometer. In Figure 4, a forest plot was used to illustrate a source-based skin elasticity meta-analysis, comparing patients receiving HC supplementation to the placebo group. Within Figure 4, subgroup A displayed a significant increase, with a Z-value of 4.24 (P < 0.0001), supported by an overall effect size (95% CI) of 0.54 (0.29, 0.79) concerning skin elasticity. Additionally, a subgroup analysis based on the duration of HC supplementation is presented in a forest plot for a source-classified meta-analysis of skin elasticity, comparing the HC-supplemented group to the placebo group. As shown in Figure 5, subgroup A revealed a significant increase, with a Z-value of 6.16 (P < 0.00001) and an overall effect size (95% CI) of 0.65 (0.44, 0.85) about skin elasticity.

**Figure 6:**
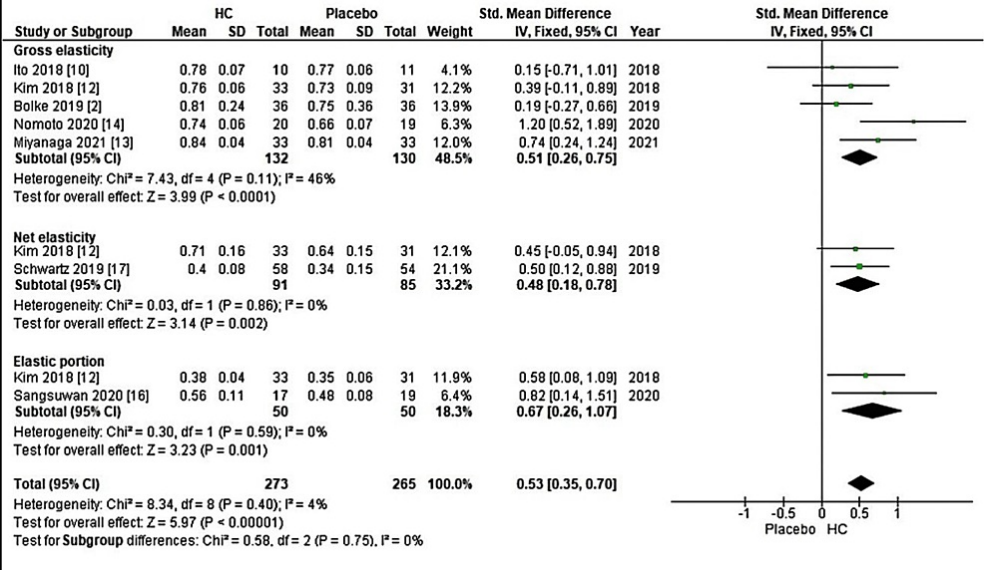
Forest plot of comparison: skin elasticity, outcome: cutometer. HC: hydrolyzed collagen This figure was created by NW, one of the authors of this article.

The final subgroup analysis aimed to assess the influence of diverse sources of HC and the corresponding measurements, which included R2 (gross elasticity), R5 (net elasticity, representing the elastic portion from relaxation to the elastic portion from suction), and R7 (elastic portion measured using a cutometer) on skin elasticity. The results of this subgroup analysis revealed a significant increase, with a Z-value of 5.97 (P < 0.00001), along with an overall effect size (95% CI) of 0.53 (0.35, 0.70) concerning skin elasticity (Figure 6).

**Figure 7:**
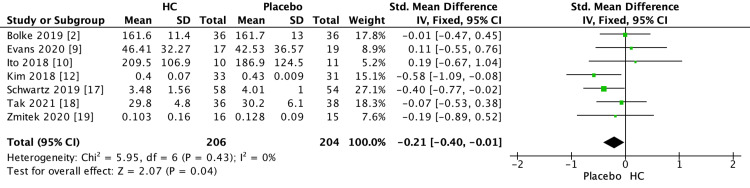
Forest plot of comparison: the comparison of wrinkles influenced by hydrolyzed collagen (wrinkles, outcome: the 6th generation VISIA skin analysis system (Canfield Imaging Systems, Parsippany, NJ)). HC: hydrolyzed collagen. This figure was created by AP, one of the authors of this article.

Subsequently, the research investigated how the source and duration of HC supplementation influence the development of wrinkles. The analysis employed a forest plot, depicted in Figure 7, to present a meta-analysis of wrinkles, comparing individuals who were administered HC supplementation with those in the placebo group. Figure 7 demonstrated a noteworthy enhancement in the HC effect, indicating a Z-value of 2.07 (P = 0.04). This finding was substantiated by an overall effect size (95% CI) of -0.21 (-0.40, -0.01) about skin wrinkles.

**Figure 8:**
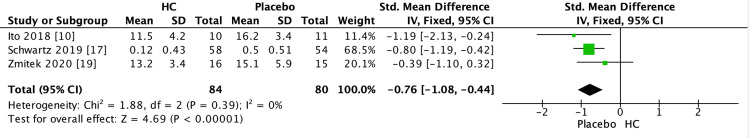
Forest plot of comparison: the comparison of transepidermal water loss influenced by hydrolyzed collagen (TEWL, outcome: corneometer). HC: hydrolyzed collagen; TEWL: transepidermal water loss. This figure was created by NA, one of the authors of this article.

Additionally, this investigation carried out an analysis to assess how the source and duration of HC supplementation impact TEWL. In Figure 8, a forest plot was utilized to depict the TEWL meta-analysis, comparing individuals who were administered HC supplementation with those in the placebo group. The findings in Figure 8 revealed a substantial increase in the HC effect, indicating a Z-value of 4.69 (P < 0.00001). This outcome was substantiated by an overall effect size (95% CI) of -0.76 (-1.08, -0.44) about skin TEWL.

**Figure 9:**
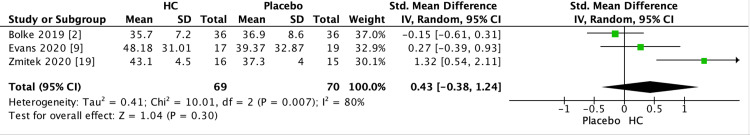
Forest plot of comparison: the comparison of skin firmness influenced by hydrolyzed collagen (skin firmness, outcome: Visual Analogue Scale (VAS) questionnaire). HC: hydrolyzed collagen. This figure was created by NS, one of the authors of this article.

Subsequently, the research analyzed how the source and duration of HC supplementation affect skin firmness. In Figure 9, a forest plot was employed to present the meta-analysis of firmness, comparing individuals who received HC supplementation with those in the placebo group. In Figure 9, the impact of HC exhibited a noteworthy enhancement, indicating a Z-value of 1.04 (P = 0.30). This improvement was substantiated by an overall effect size (95% CI) of 0.43 (-0.38, 1.24) concerning the firmness of the skin.

**Figure 10:**
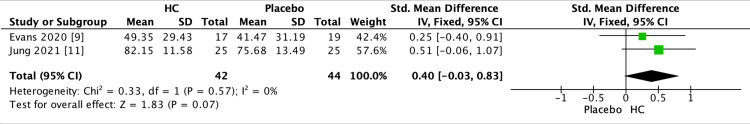
Forest plot of comparison: the comparison of skin radiance influenced by hydrolyzed collagen (skin radiance, outcome: Visual Analogue Scale (VAS) questionnaire). HC: hydrolyzed collagen. This figure was created by SY, one of the authors of this article.

Moreover, this investigation carried out an analysis to assess how the source and duration of HC supplementation impact skin radiance. Figure 10 utilized a forest plot to portray the radiance meta-analysis, comparing individuals who were administered HC supplementation with those in the placebo group. In Figure 10, the HC effect demonstrated a notable improvement, indicating a Z-value of 1.83 (P = 0.07). This enhancement was substantiated by an overall effect size (95% CI) of 0.40 (-0.03, 0.83) about skin radiance.

Bias

**Figure 11:**
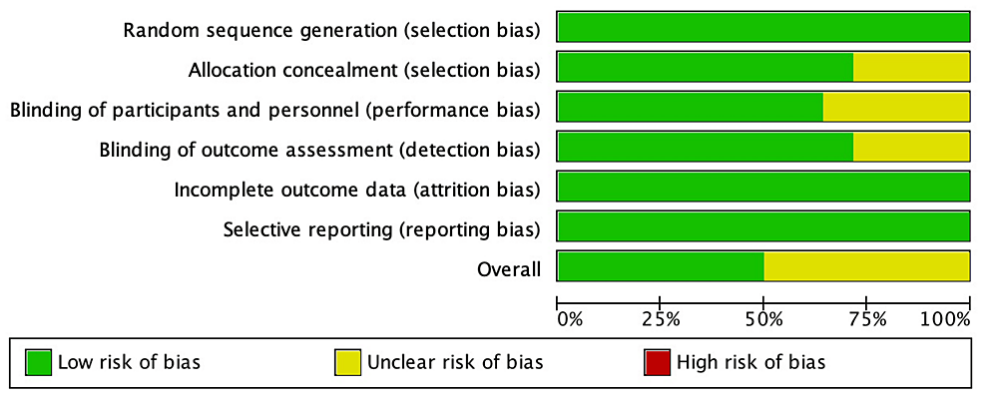
Risk of bias graph. This figure was created by FN, one of the authors of this article.

**Figure 12:**
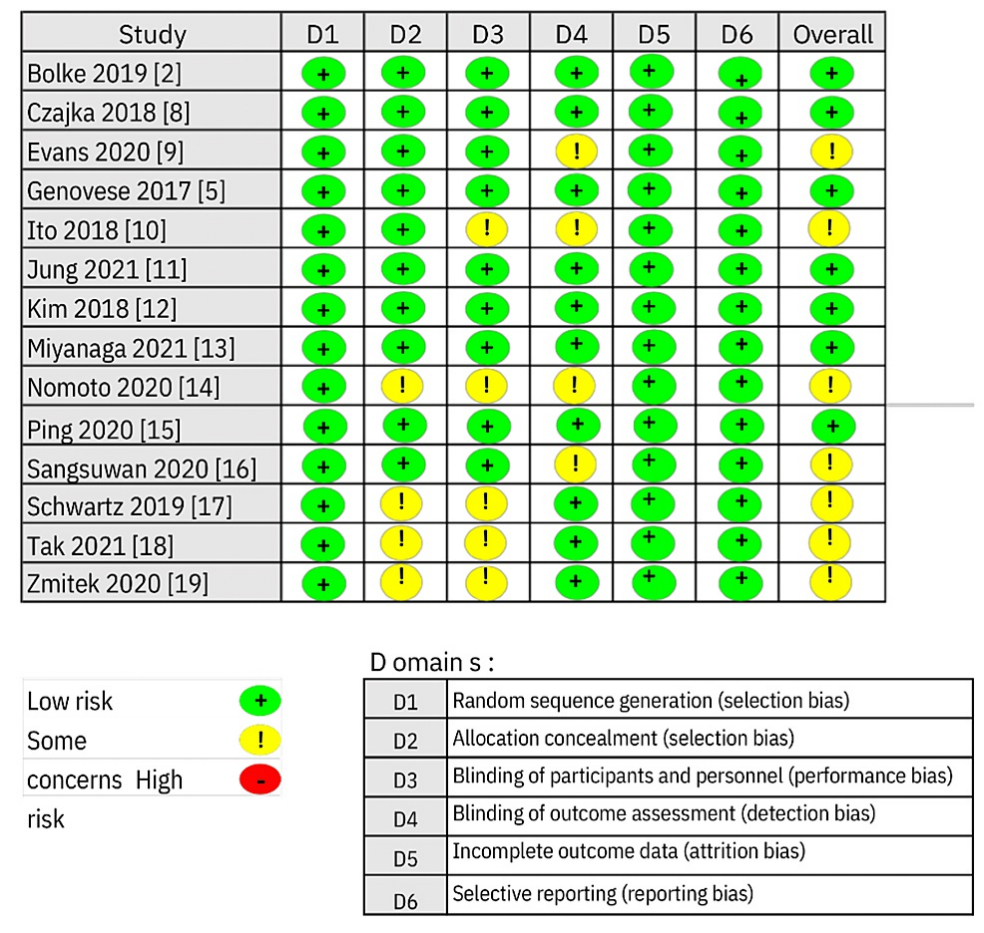
Risk of bias summary. This figure was created by FN, one of the authors of this article.

The quality of the risk of bias (RoB) was evaluated using the RoB-2 tool, with an assessment made at both the domain and study levels. The domain-level RoB assessment revealed a low RoB in seven RCTs and an unclear RoB in another seven RCTs, as depicted in Figure 11. Meanwhile, at the study level, the RoB was observed in terms of the blindness of participants and researchers in studies conducted by Ito et al. (2018) [10], Nomoto et al. (2020) [14], Schwartz et al. (2019) [17], Tak et al. (2021) [18], and Zmitek et al. (2020) [19], as illustrated in Figure 12.

Publication Bias

**Figure 13:**
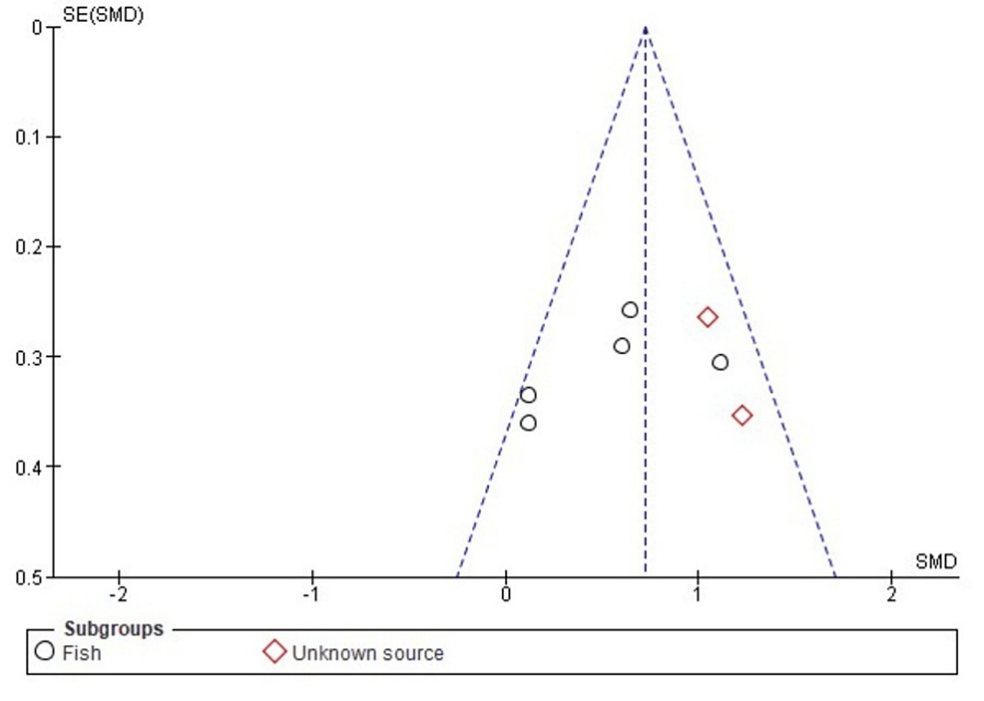
Funnel plot of comparison: skin hydration, outcome: source. This figure was created by NA, one of the authors of this article.

**Figure 14:**
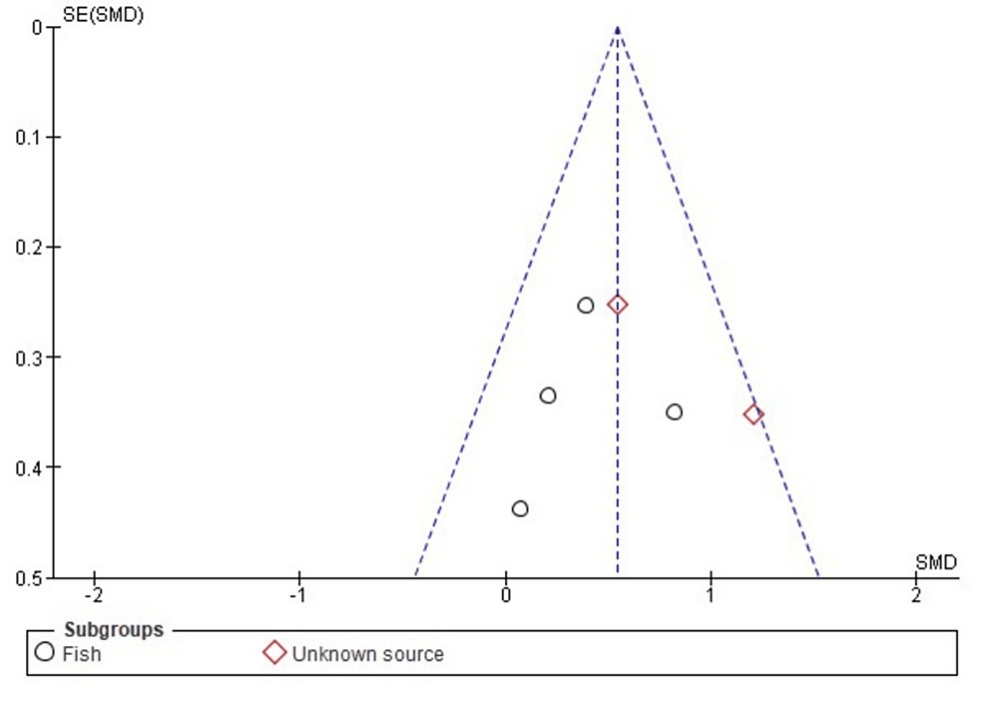
Funnel plot of comparison: skin elasticity, outcome: source. This figure was created by NA, one of the authors of this article.

A visual assessment of the funnel plot, as depicted in Figures 13, 14, shows a balanced distribution, suggesting that the variations in the data primarily stem from sample differences rather than publication bias. These plots depict data with the vertical axis representing the standard error, offering an estimate of each study's sample size, with more extensive studies at the top and smaller studies at the bottom. The horizontal axis expansion reflects the strength and effect size of the studies analyzed.

The symmetrical arrangement of data points in the funnel plot assures that the findings are not influenced by selective publication of studies based on their results. This symmetry indicates that both large and small studies have been considered, and their distribution aligns with what one would expect in the absence of bias. Therefore, the lack of any noticeable asymmetry in the funnel plot further underscores the reliability of the study's conclusions, reinforcing the robustness of the analysis.

Discussion

The Effect of Hydrolyzed Collagen on Skin Rejuvenation

The dermal structure consists of two distinct layers: the adventitial dermis, characterized by thin collagenous fibers, and the reticular dermis, which features thick, coarse collagen bundles [1,20,21]. Skin elasticity is a fundamental aspect of skin health, primarily governed by the composition and structure of the dermal [21-23]. The extracellular matrix (ECM) comprises crucial elements like collagen, elastic fibers, and hyaluronic acid (HA), with collagen being a key contributor to maintaining skin elasticity. Collagen constitutes a significant portion of the dry weight of the typical human skin dermis, exceeding 70% [24]. Elastic fibers, another integral part of the ECM, consist of an inner core composed of crosslinked elastin surrounded by outer layers of fibrils and microfibrils [21-23]. The natural aging process, influenced by both intrinsic and extrinsic factors, can lead to a reduction in skin elasticity. Chronic exposure to sunlight, often referred to as photoaging, can induce significant alterations in the skin, including a decrease in collagen and elastic fibers [22]. Over time, in conjunction with reduced HA synthesis, this can result in the formation of wrinkles, dryness, and a decrease in skin elasticity [5].

Our meta-analysis of 14 studies on oral administration of HC after four weeks proved it improves skin hydration [2,9,11-15,19], elasticity [2,5,8-10,12-14,16,17], wrinkles [2,9,10,12,17-19], TEWL [10,17,19], firmness [2,9,19], and radiance [9,11].

Typically, sources of HC include fish, chicken, pork, and cows. Fish-derived collagen has recently been a popular substitute because of its reduced risk of disease transmission and environmental effects. Moreover, the structure and bioavailability of collagen and collagen peptides from marine fish are quite similar to those of humans across the gastrointestinal barrier [9]. Of the 14 studies that conducted a meta-analysis, it was found that several researchers used HC sourced from fish [8-12,15,16,18,19].

The HC source used in Czajka et al.'s study was a liquid nutraceutical sold under the brand name GOLD COLLAGEN® ACTIVE, a liquid food supplement manufactured by MINERVA Research Labs (London, UK) that included hydrolyzed fish collagen, antioxidants, chondroitin sulfate, glucosamine, L-carnitine, vitamins, and minerals. In the intervention group, there was an improvement in skin texture and elasticity as measured by the SkinLab USB Elasticity Module (DermaLab® Series, Cortex Technology, Hadsund, Denmark) and incisional biopsy (4.0 mm punch) on the inner forearm. Histological analysis of skin biopsies showed positive changes in skin architecture, with decreased solar elastosis and increased collagen fiber organization in the test product group [8].

Evans et al. studied women between the ages of 45 and 60 years utilizing an HC source of hydrolyzed marine collagen (manufactured by Vinh Wellness Collagen, Cao Lãnh, Vietnam). The VISIA skin analysis system (Canfield Imaging Systems, Parsippany, NJ) is used for wrinkle evaluation, the Cutometer® tool is used for measuring elasticity, and the Skin Quality, Visual Analogue Scale, is used for measuring self-reported appearance. The study's findings favor using fish-derived HC to enhance skin health in older people [9].

Ito et al. (2018) examined the effect of a combined supplement containing collagen peptides and ornithine (CPO) derived from fish in improving skin conditions by increasing plasma levels of growth hormone and insulin-like growth factor-1 (IGF-1). It was proven that skin elasticity, wrinkles, plasma growth hormone, and IGF-1 levels improved in the CPO group and decreased TEWL [10].

Jung et al. investigated whether oral intake of enzymatically degraded AP collagen peptide (APCP) could improve skin moisture and barrier function by assessing changes in ceramide and natural moisturizing factor (NMF) content in SC after consumption of APCP to develop functional skin foods. APCPs were provided by Aestura Corp. (Seoul, Korea) and were manufactured by the enzymatic degradation of gelatin derived from the scales of *Nemipterus virgatus.* The APCPs contained >15% tripeptides and 3% Gly-Pro-Hyp. It is proven that the intake of APCP increases skin moisture and the content of ceramide and NMF in the stratum corneum (SC), thereby improving the skin barrier function [11].

Kim et al. investigated the effects on hydration, wrinkles, and skin elasticity using low molecular weight collagen peptides (LMWCP) 1000 mg once daily for 12 weeks. The LMWCP used herein (obtained from Newtree, Seongnam, Korea) was a collagen hydrolysate (CH) obtained from the sutchi catfish’s skin (*Pangasius hypophthalmus*), with >15% tripeptide content including 3% Gly-Pro-Hyp. The results showed that these three parameters increased significantly compared to the control group [12].

Ping et al. investigated the efficacy of the synergistic effect of fish collagen and Djulis (Chenopodium formosanum Koidz) for improving skin parameters for eight weeks. The collagen composition used has a brand Diva Luxe collagen drink (50 g; main ingredient: 81% water, 11% fish collagen (extracted from *Pangasius hypophthalmus*, *Oreochromis niloticus*, *Clarias gariepinus*, *Gadus morhua*, *Ictalurus furcatus*, and *Melanogrammus aeglefinus*), 3% apple juice, 2% Djulis extract). This clinical study demonstrates the improvements in hydration, brightness, crow's feet, texture, wrinkles, pores, surface spots, and collagen content in the skin [15].

Sangsuwan et al. evaluated the effect of CH ingestion on skin elasticity for four weeks. The CH used in this study was derived from fish scale and skin manufactured by Nippi Peptide FCP-EX in a 5-gram powder formulation per packet (Nippi, Shizuoka, Japan) and maltodextrin (Zhucheng Dongxiao Biotechnology, Zhucheng City, Shandong, China). They demonstrated significant improvement in skin elasticity in sun-exposed areas after four weeks of ingestion of marine CH. The progress of elasticity remained four weeks after discontinuation of study agents [16].

Tak et al. tested the effect of collagen tripeptide (CTP) and adjusting for climate change on skin properties in middle-aged women. CTP was prepared from the skin of Nile tilapia by the digestion method using collagenase from non-pathogenic bacteria of the genus Bacillus. Compared with the control group, TEWL was reduced more in the CTP group, even after adjustment for humidity, temperature, and ultraviolet A (UVA) in the region and increased skin moisture [18].

Zmitek et al. investigated the effects of a liquid food supplement, characterized by a combination of water-soluble coenzyme Q10 (Q10Vital®, Šenčur, Slovenia) and hydrolyzed fish collagen, on dermal density and other skin parameters compared to placebo. They observed improved dermis density, reduced periorbital wrinkle area and total wrinkle score, and improved skin smoothness. On the other hand, changes in skin hydration, dermis thickness, TEWL, and viscoelasticity were not significant [19].

Even though the collagen used comes from fish, the type of fish is different, and some researchers also combine it with other active ingredients. Overall, all studies show significant results in improving skin conditions. Thus, improvements in the skin can be caused by the influence of not only collagen alone.

Other researchers use HC derived from other than fish products. Four other studies used collagen peptides but did not clearly state where they came from [2,5,13,14]. One study used collagen derived from chicken [17].

An oral ampoule containing a combination of 2.5 g of collagen peptides, acerola fruit extract, vitamin C, zinc, biotin, and the original vitamin E complex marketed under the brand name ELASTEN® (made by QUIRIS Healthcare, Gütersloh, Germany) was used in research by Bolke et al. In the intervention group, there was an increase in skin hydration, skin elasticity, and skin density. Furthermore, the study found that these positive effects persisted after four weeks without further supplementation [2].

Genovese et al. employ HC derived from bioactive collagen peptides and antioxidants; the product is called GOLD COLLAGEN ® FORTE and is taken orally. UK-based MINERVA Research Labs manufacture it. The SkinLab USB Elasticity Module (DermaLab ® Series, Cortex Technology, Hadsund, Denmark) was used to evaluate the skin elasticity measurement results. A right buttock surface incisional biopsy (punch 4.0 mm) assesses the skin's structure, namely, the collagen and elastin fibers [5].

Miyanaga et al. investigated the effect of orally ingested collagen peptides (CPs) on skin conditions and elucidated their mechanism of action. CPs (product name: FCP-Z; average molecular weight: 5 kDa) were purchased from Nippi Co., Ltd (Tokyo, Japan). The oral ingestion of CP increased the water content in the SC and epidermis and decreased TEWL. Furthermore, the NMF level in the SC was increased. However, skin elasticity and skin thickness remained unchanged [13].

Nomoto et al. investigated the effect of an oral nutrition supplement containing collagen peptides V CRESC CP10 (Nutri Co., Ltd, Yokkaichi, Japan) on SC hydration and skin elasticity. The study result was that mean SC hydration significantly increased from 43.7 at baseline to 51.7 at postintervention week eight in the intervention group (P = 0.001). Differences in skin elasticity from baseline were significant at postintervention week six (P = 0.026) and week eight (P = 0.049) [14].

Schwartz et al. investigated the correlation that existed between the effects of a collagen dietary supplement containing 500 mg BioCell Collagen, a chicken sternal cartilage-derived dietary ingredient composed of a naturally occurring matrix of HC type-II (≥300 mg), chondroitin sulfate (≥100 mg), and HA (≥50 mg), and changes associated with skin aging. It significantly reduced facial lines and wrinkles and crow's feet lines and wrinkles, increased skin elasticity and cutaneous collagen content by 12%, improved indicators associated with a youthful skin appearance based on visual grading and wrinkle width, and decreased skin dryness and erythema [17].

Within the realm of skincare products, the challenge often lies in the ability of topically applied creams, lotions, and serums to deeply penetrate the skin's layers and provide long-lasting effects on the skin's aging processes [14]. However, the development of highly bioavailable and bioactive short-chain nutritional collagen peptides has successfully targeted the dermis, a pivotal skin layer responsible for both collagen synthesis and skin rejuvenation. This trial has reaffirmed the effectiveness of collagen supplementation in enhancing skin density and elasticity [12,23]. Collagen supplements can play a valuable role in sustaining skin health [25].

Mechanism of Hydrolyzed Collagen

When HC is ingested, it travels through the bloodstream in the form of dipeptides (e.g., Gly-Pro and Pro-Hyp) and tripeptides (e.g., Gly-Pro-Hyp) [20]. These collagen-derived peptides are distributed to various tissues, including the skin, where they accumulate as peptides or amino acids (AAs). This accumulation triggers a chemotactic response in skin fibroblasts, increasing their activity [20,26].

Dipeptides containing Hyp, like Pro-Hyp, play a particularly significant role when absorbed by the skin [5,20]. They promote the growth of dermal fibroblasts and enhance the production of HA, resulting in increased moisture levels in the outermost skin layer, the SC, thus improving skin hydration [6]. Additionally, collagen peptides support collagen synthesis at both the mRNA and protein levels, forming robust collagen fibrils that strengthen the skin's natural barrier, ultimately enhancing skin elasticity [19].

Furthermore, the intake of collagen peptides stimulates the production of filaggrin, a crucial element in restoring the skin's barrier function. Filaggrin increases the levels of AAs and amino acid derivatives (AADs), essential components of the skin's NMF within the SC, further contributing to skin hydration [4,20]. In a comprehensive study, the consumption of collagen peptides (APCP) was found to enhance skin barrier function and skin hydration, as evidenced by a significant increase in the total content of AAs and AADs in the SC in the APCP group compared to the placebo group. This demonstrates that APCP intake enhances skin moisture and effectively maintains it by elevating the levels of NMF components, such as AAs and AADs, within the SC [20].

Based on the research reviewed, no one reported any side effects from consuming collagen. HC supplements are commonly derived from bovine, marine animals, and chicken. This means that if the consumers are allergic to beef, fish, or poultry, they could have a reaction after consuming collagen supplements. Fujimoto et al. (2016) reported a patient with atopic dermatitis who experienced episodes of anaphylaxis after ingestion of a dietary supplement that contained hydrolyzed fish collagen [27].

In summary, the mechanism by which HC improves skin elasticity and hydration involves the stimulation of collagen synthesis, an increase in moisture content within the SC, and the reinforcement of the skin's natural barrier. These combined effects contribute to overall skin health and vitality, making collagen supplements effective in preserving rejuvenation and maintaining a well-nourished complexion. HC is more easily absorbed than other types. It is generally available in capsule or powder form, which can be added to drinks, soups, and even other foods. Research shows a safe and effective dose for skin rejuvenation is 300 mg to 5 grams of HC peptides daily. Collagen preparations can be combined with other active ingredients. HC will provide benefits if consumed for more than four weeks. Collagen is a safe, non-toxic daily supplement for healthy individuals, and most people will not experience adverse side effects. However, some people report symptoms, such as an unpleasant taste or other stomach complaints. Although rare, people can be allergic to the source of collagen they consume.

Limitations

Limitations of this review may result from the inclusion of several studies that showed significant heterogeneity, mainly due to differences in the composition of the supplements used based on the HC source; some use products from various fish, and other research combines with active ingredients. The administration dose also varies, and the duration of administration is different. The effects will differ if given to the study population of women, men, teenagers, adults, and post-menopausal women. The tools used to measure the parameters studied also vary. Variability may influence comparisons in terms of interventions and outcomes. The methodology was applied to verify the results. However, it is essential to note that in all the studies reviewed, oral consumption of collagen hydrolyzate and HC peptides has shown effectiveness in reducing signs of skin aging and is safe to consume, with potential long-term benefits. What is also essential for research to explore is the participants' lifestyle. Individuals who lead a healthy lifestyle, characterized by a balanced diet and adequate hydration, may experience more marked and rapid improvements in skin appearance through collagen supplementation compared to those with less healthy lifestyle habits.

## Conclusions

The review results show that HC positively affects skin elasticity and hydration, thereby contributing to overall skin rejuvenation. The forest plots in the study visually depicted the effect of HC supplementation regarding skin elasticity and hydration compared to the placebo group. It should be noted that subgroup analysis showed significant differences in results based on different HC sources for skin elasticity and moisture. Although these findings are promising, it is essential to conduct large-scale, RCTs involving healthy living habits as one of the factors that serve as indicators for assessing the success of oral collagen supplements in rejuvenating the skin.

## Appendices

Study selection by keywords

Cochrane Central Register of Controlled Trials (CENTRAL)

**Table 2:** The Cochrane Central Register of Controlled Trials (CENTRAL). This table was created by one of the authors of this article.

No.	Search	Result
#1	"collagen" OR "hydrolyzed collagen"	10287
#2	oral supplementation	10778
#3	"skin rejuvenation" OR "skin aging"	1422
#4	hydration skin	1410
#5	elasticity skin	1001
#6	#1 AND #2 AND #3 AND #4 AND #5	32
Total: 32		

PubMed

**Table 3:** PubMed. This table was created by one of the authors of this article.

No.	Search	Result
#1	"collagen" OR "hydrolyzed collagen"	4827
#2	oral supplementation	4709
#3	"skin rejuvenation" OR "skin aging"	1106
#4	hydration skin	472
#5	elasticity skin	442
#6	#1 AND #2 AND #3 AND #4 AND #5	6
Total: 6		

Google Scholar

(((Hydrolyzed collagen [MeSH Terms]) AND (Skin rejuvenation OR skin aging [MeSH Terms])) AND (Skin elasticity[MeSH Terms])) AND (Skin hydration[MeSH Terms]))

Total: 6950

ScienceDirect

(((Hydrolyzed collagen [MeSH Terms]) AND (Skin rejuvenation OR skin aging [MeSH Terms])) AND (Skin elasticity[MeSH Terms])) AND (Skin hydration[MeSH Terms]))

Total: 159

## References

[1] Jafari H, Lista A, Siekapen MM, Ghaffari-Bohlouli P, Nie L, Alimoradi H, Shavandi A (2020). Fish collagen: extraction, characterization, and applications for biomaterials engineering. Polymers (Basel).

[2] Bolke L, Schlippe G, Gerß J, Voss W (2019). A collagen supplement improves skin hydration, elasticity, roughness, and density: results of a randomized, placebo-controlled, blind study. Nutrients.

[3] Di Cerbo A, Laurino C, Palmieri B, Iannitti T (2015). A dietary supplement improves facial photoaging and skin sebum, hydration and tonicity modulating serum fibronectin, neutrophil elastase 2, hyaluronic acid and carbonylated proteins. J Photochem Photobiol B.

[4] Aldag C, Nogueira Teixeira D, Leventhal PS (2016). Skin rejuvenation using cosmetic products containing growth factors, cytokines, and matrikines: a review of the literature. Clin Cosmet Investig Dermatol.

[5] Genovese L, Corbo A, Sibilla S (2017). An insight into the changes in skin texture and properties following dietary intervention with a nutricosmeceutical containing a blend of collagen bioactive peptides and antioxidants. Skin Pharmacol Physiol.

[6] Lupu MA, Gradisteanu Pircalabioru G, Chifiriuc MC, Albulescu R, Tanase C (2020). Beneficial effects of food supplements based on hydrolyzed collagen for skin care (review). Exp Ther Med.

[7] Maia Campos PMBG, Melo MO, Calixto LS, Fossa MM (2015). An oral supplementation based on hydrolyzed collagen and vitamins improves skin elasticity and dermis echogenicity: a clinical placebo-controlled study. Clin Pharmacol Biopharm.

[8] Czajka A, Kania EM, Genovese L, Corbo A, Merone G, Luci C, Sibilla S (2018). Daily oral supplementation with collagen peptides combined with vitamins and other bioactive compounds improves skin elasticity and has a beneficial effect on joint and general wellbeing. Nutr Res.

[9] Evans M, Lewis ED, Zakaria N, Pelipyagina T, Guthrie N (2021). A randomized, triple-blind, placebo-controlled, parallel study to evaluate the efficacy of a freshwater marine collagen on skin wrinkles and elasticity. J Cosmet Dermatol.

[10] Ito N, Seki S, Ueda F (2018). Effects of composite supplement containing collagen peptide and ornithine on skin conditions and plasma IGF-1 levels—a randomized, double-blind, placebo-controlled trial. Mar Drugs.

[11] Jung K, Kim SH, Joo KM (2021). Oral intake of enzymatically decomposed AP collagen peptides improves skin moisture and ceramide and natural moisturizing factor contents in the stratum corneum. Nutrients.

[12] Kim DU, Chung HC, Choi J, Sakai Y, Lee BY (2018). Oral intake of low-molecular-weight collagen peptide improves hydration, elasticity, and wrinkling in human skin: a randomized, double-blind, placebo-controlled study. Nutrients.

[13] Miyanaga M, Uchiyama T, Motoyama A, Ochiai N, Ueda O, Ogo M (2021). Oral supplementation of collagen peptides improves skin hydration by increasing the natural moisturizing factor content in the stratum corneum: a randomized, double-blind, placebo-controlled clinical trial. Skin Pharmacol Physiol.

[14] Nomoto T, Iizaka S (2020). Effect of an oral nutrition supplement containing collagen peptides on stratum corneum hydration and skin elasticity in hospitalized older adults: a multicenter open-label randomized controlled study. Adv Skin Wound Care.

[15] Lin P, Alexander RA, Liang CH (2021). Collagen formula with Djulis for improvement of skin hydration, brightness, texture, crow's feet, and collagen content: a double-blind, randomized, placebo-controlled trial. J Cosmet Dermatol.

[16] Sangsuwan W, Asawanonda P (2021). Four-weeks daily intake of oral collagen hydrolysate results in improved skin elasticity, especially in sun-exposed areas: a randomized, double-blind, placebo-controlled trial. J Dermatolog Treat.

[17] Schwartz SR, Hammon KA, Gafner A, Dahl A, Guttman N, Fong M, Schauss AG (2019). Novel hydrolyzed chicken sternal cartilage extract improves facial epidermis and connective tissue in healthy adult females: a randomized, double-blind, placebo-controlled trial. Altern Ther Health Med.

[18] Tak YJ, Shin DK, Kim AH (2020). Effect of collagen tripeptide and adjusting for climate change on skin hydration in middle-aged women: a randomized, double-blind, placebo-controlled trial. Front Med (Lausanne).

[19] Žmitek K, Žmitek J, Rogl Butina M, Pogačnik T (2020). Effects of a combination of water-soluble coenzyme Q10 and collagen on skin parameters and condition: results of a randomised, placebo-controlled, double-blind study. Nutrients.

[20] Campos LD, Santos Junior VA, Pimentel JD, Carregã GL, Cazarin CB (2023). Collagen supplementation in skin and orthopedic diseases: a review of the literature. Heliyon.

[21] Shin JW, Kwon SH, Choi JY, Na JI, Huh CH, Choi HR, Park KC (2019). Molecular mechanisms of dermal aging and antiaging approaches. Int J Mol Sci.

[22] Tanaka M, Yamamoto Y, Misawa E (2016). Effects of aloe sterol supplementation on skin elasticity, hydration, and collagen score: a 12-week double-blind, randomized, controlled trial. Skin Pharmacol Physiol.

[23] Song H, Zhang L, Luo Y, Zhang S, Li B (2018). Effects of collagen peptides intake on skin ageing and platelet release in chronologically aged mice revealed by cytokine array analysis. J Cell Mol Med.

[24] Yoon HS, Cho HH, Cho S, Lee SR, Shin MH, Chung JH (2014). Supplementating with dietary astaxanthin combined with collagen hydrolysate improves facial elasticity and decreases matrix metalloproteinase-1 and -12 expression: a comparative study with placebo. J Med Food.

[25] Kim JE, Kim HS (2019). Microbiome of the skin and gut in atopic dermatitis (Ad): understanding the pathophysiology and finding novel management strategies. J Clin Med.

[26] Inoue N, Sugihara F, Wang X (2016). Ingestion of bioactive collagen hydrolysates enhance facial skin moisture and elasticity and reduce facial ageing signs in a randomised double-blind placebo-controlled clinical study. J Sci Food Agric.

[27] Fujimoto W, Fukuda M, Yokooji T, Yamamoto T, Tanaka A, Matsuo H (2016). Anaphylaxis provoked by ingestion of hydrolyzed fish collagen probably induced by epicutaneous sensitization. Allergol Int.

